# The Effects of Football Practice on Nutritional Status and Body Composition in Children: A Systematic Review and Meta-Analysis

**DOI:** 10.3390/nu13082562

**Published:** 2021-07-26

**Authors:** Antonio Hernandez-Martin, Jorge Garcia-Unanue, Alejandro Martínez-Rodríguez, Samuel Manzano-Carrasco, Jose Luis Felipe, Maria Jose Carvalho, Leonor Gallardo, Javier Sanchez-Sanchez

**Affiliations:** 1 IGOID Research Group, Physical Activity and Sport Sciences Department, University of Castilla-La Mancha, 45004 Toledo, Spain; Antonio.HMartinSan@uclm.es (A.H.-M.); Samuel.Manzano@uclm.es (S.M.-C.); Leonor.Gallardo@uclm.es (L.G.); 2 Analytical Chemistry, Nutrition and Food Science Department, University of Alicante, 03690 Alicante, Spain; amartinezrodriguez@gcloud.ua.es; 3 School of Sport Sciences, Universidad Europea de Madrid, 28670 Madrid, Spain; joseluis.felipe@universidadeuropea.es (J.L.F.); javier.sanchez2@universidadeuropea.es (J.S.-S.); 4 Centre of Research, Education, Innovation and Intervention in Sport (CIFI2D), Faculty of Sport, University of Porto, 4099-002 Porto, Portugal; mjc@fade.up.pt

**Keywords:** body composition, football exercise, child athletes

## Abstract

Dietary patterns, exercise, sport, and physical activity have been shown to improve body composition in children. This systematic review with meta-analysis analyzed the effects of practicing football on body composition (fat mass, lean body mass, and bone mineral content) in children. An initial search in PubMed, Web of Science, and SPORTDiscus was carried out in April 2021 to identify relevant articles. Inclusion criteria required children up to 12 years of age with a minimum football intervention duration of 10 weeks. Methodological quality of the articles was evaluated using the PEDro scale. Between the 1803 articles originally founded, only 14 articles were included in the meta-analysis. A total of 1643 subjects between the 14 studies were identified. The review and meta-analysis were conducted according to the Preferred Reporting Items for Systematic Reviews and Meta-Analyses (PRISMA) guidelines and used Review Manager and Full Meta-Analysis software. The results between the control and experimental groups showed significantly better lean body mass and fat mass values in the experimental group (*p* < 0.05). Football practice was positively associated with increases in lean body mass (mean difference of 1.55; 95% CI, 0.96, 2.15), decreases in fat mass (mean difference of −0.81; 95% CI, −1.49, −0.13), and increases in whole body bone mineral content (mean difference of 117.68; 95% CI, 83.69, 151.67). In conclusion, the results of this systematic review with meta-analysis suggest that football positively affects body composition in children. However, further research is needed to confirm the results for bone mineral content.

## 1. Introduction

Inadequate dietary patterns and sedentary lifestyles have led to an increase in the percentage of cardiovascular and osteogenic diseases in all age groups of the population. The percentage of overweight children has increased exponentially in recent years [1], and currently, approximately 100 million children and adolescents are overweight [2].

The overweight and obesity, due to an excessive gain of adipose weight, is related to unhealthy behaviors, and may be accelerated by them [3]. In this sense, dietary patterns, including the meals and nutrients distribution, have great influence. Sedentarism and time spent in front of a screen reduces engagement in moderate-to-vigorous physical activity. Regarding dietary patterns, unhealthy behaviors can expectedly cause a decrease in the intake of healthy foods and an increase in the consumption of unhealthy foods [4]. Promoting healthy lifestyles, reducing sedentary time, and increasing physical activity may help in attaining successful nutritional status, due to the energy balance in children. [5].

Childhood obesity is associated with metabolic factors such as diabetes or hypertension in adulthood [6]. Another long-term consequence of childhood sedentary lifestyles is osteoporosis, which is characterized by low bone mineral density. This disease is quite common among Caucasian and Asian women and elderly people [7]. 

The parameter associated with these illnesses is excess levels of fat mass. According to the World Health Organization (WHO), children are considered overweight >+1SD (Z-scores (BMI in kg/m^2^)) and obese >+2SD (Z-scores (BMI in kg/m^2^)); the values for overweightness and obesity in children according to age are as follows: 9 years: >17.9 kg/m^2^ and >20.5 kg/m^2^, respectively; 10 years: >18.5 kg/m^2^ and >21.4 kg/m^2^, respectively; 11 years: >19.2 kg/m^2^ and >22.5 kg/m^2^, respectively; and 12 years >19.9 kg/m^2^ and >23.6 kg/m^2^, respectively. The values for overweightness and obesity in girls according to age are as follows: 9 years: >18.3 kg/m^2^ and >21.5 kg/m^2^, respectively; 10 years: >19.0 kg/m^2^ and >22.6 kg/m^2^, respectively; 11 years: >19.9 kg/m^2^ and >23.7 kg/m^2^, respectively; and 12 years: >20.8 kg/m^2^ and >25.0 kg/m^2^, respectively. The first step to address this problem is the accurate diagnosis and monitoring of excess fat [8]. Typically, research has analyzed body composition by measuring weight, height, nutritional status, and body mass index (BMI). 

However, BMI has inherently low specificity [9] and appropriate body composition measurements is recommended [10]. Accurate assessments of fat mass and fat-free mass can increase the capacity to identify the effects of adiposity excess, as well as the effectiveness of interventions to reduce obesity levels. Given the detrimental effects on physical and mental health, there is an international call to develop strategies to prevent and manage pediatric overweight and obesity [8]. 

Childhood is a decisive period to avoid these diseases, and, for this reason, the American College of Sports Medicine has established strategies and recommendations based on physical activity to prevent obesity and osteoporosis [11]. Long-term physical activity leads to a reduction in cardiovascular diseases associated with obesity [12,13]. On the other hand, 26% of adult bone mineral content is met between the ages of 12 and 14 [14]. Therefore, using physical activity for proper bone mass accumulation during growth may be essential to reduce the risk of bone fractures in adulthood and old age [15].

Nevertheless, not all physical activities or sports have adequate characteristics to generate improvements in body composition [16], nor the same adherence in the child population. Team sports, such as football, increase motivation and adherence in the child population [17]. Football is played by children in 7 vs. 7, 8 vs. 8. or small-sided matches (3 vs. 3, 4 vs. 4) [18]. The sport has specific qualities and characteristics to prevent or reduce obesity, as the energy expenditure is high due to the high aerobic component [19]. It has been shown that the aerobic demands of football in children average between 70–90% of their heart rate max (hrmax), which leads to higher fat oxidation during exercise and greater fat loss compared to other activities of lower physical intensity [18]. Football also includes multitude high-speed actions, sprints, turns, jumps, and shots [20], and has been demonstrated that the practice of football at an early age produces benefits in bone development, due to the impacts generated during its practice [21]. This can lead to increased levels of bone mineral content and bone mineral density during growth [22]. 

Numerous studies have found that children under the age of 12 who participated in football had better exercise capacity, lower resting heart rate, and higher muscle mass than children who did not participate in recreational sports [23,24]. However, not all football interventions in children produce the same effects on body composition. Depending on the duration, intensity, and physical condition of the children, different methods of assessing body composition, and the experience of the coaches, can contribute to heterogeneous results. Therefore, the aim of this systematic review and meta-analysis was to investigate and analyze the effects of football practice on body composition—fat-free mass, fat mass, and bone mineral content (BMC)—in children. The main hypothesis of this study is that those children who undertake a football program with a minimum frequency of 2 days per week will have improved body composition compared to those who do not undertake any intervention.

## 2. Materials and Methods

### 2.1. Experimental Approach to the Problem

This study was conducted according to the Preferred Reporting Items for Systematic Reviews and Meta-Analyses (PRISMA) [25]. A search strategy was developed to identify researches that evaluated the effect of football training in children on body composition, bone development, and maximal oxygen uptake. The search was registered in PROSPERO. The systematic search was conducted in different online databases: PubMed (entire database), SPORTDiscus, and Web of Science (entire database) from 1 January 2006 to 17 April 2021. The search terms employed were: (‘soccer’ OR ‘football’) AND (‘children OR child’) AND (‘fat mass’ OR ‘lean body mass’ OR ‘bone mineral content’).

### 2.2. Elegibility Criteria

The studies included in the analysis were from original research articles that had to meet the following criteria: (1) participants were boys and girls up to 12 years old, playing football or not; (2) there was a football intervention with a minimum of 10 weeks; (3) the outcome measures were markers related to body composition (fat mass, lean body mass, BMC); (4) healthy or obese population; (5) publication in English; (6) peer-reviewed articles; (7) all testing conditions adhered to standard procedures similarly employed at baseline and following the intervention; and (8) published articles.

### 2.3. Data Extraction and Quality Assessment

The following variables were abstracted into a preformatted spreadsheet: authors, year of publication, and characteristics of study participants (*n*, age, % females, sport, BMI). Data extraction, quality assessment, and risk of bias were performed independently and in duplicate by two investigators (A.M.) and (J.S.), applying the PEDro Scale according to previous research [26]. Discrepancies were solved by consensus consulting with an independent third reviewer (L.G.) in accordance with the Cochrane Collaboration guidelines [27].

### 2.4. Data Synthesis and Analysis

A data sheet to record the most relevant information of the included researches was prepared with the following variables: authors, year, study population, intervention groups, existence of a control group, research objective and hypothesis, tools used for data collection, and relevant findings. The meta-analysis was performed to determine the effect size of football training on markers of body composition and osteogenic development, using Review Manager software (RevMan 5.3, Cochrane Collaboration, Oxford, UK) and Comprehensive Meta-Analysis software (Version 2, Biostat, Englewood, NJ, USA). 

To compare different variables, the effect of football training was calculated by the difference in the variables of body composition before football practice, while to compare the change in the football group in each variable, the values before and after the football intervention were analyzed. Each difference in the mean was weighted according to the inverse variance method [28]. Mean differences were standardized, dividing the values with their corresponding standard deviation. The standardized mean difference (SMD) for each variable was combined with a random effects model [29]. The confidence interval (95% CI) was calculated to identify the magnitude of the changes.

Articles were divided into groups according to the specific variable to be analyzed (fat mass, lean body mass, BMC) between the experimental group and the control group, and between the experimental group before and after the intervention. Heterogeneity between studies was evaluated using I^2^ statistics, so values of <25, 50 and 75 were considered to indicate low, moderate, and high heterogeneity, respectively. Publication bias was assessed by estimating the funnel plot asymmetry using the Egger test [30]. A *p*-value of less than 0.05 was considered statistically significant.

## 3. Results

**Figure 1:**
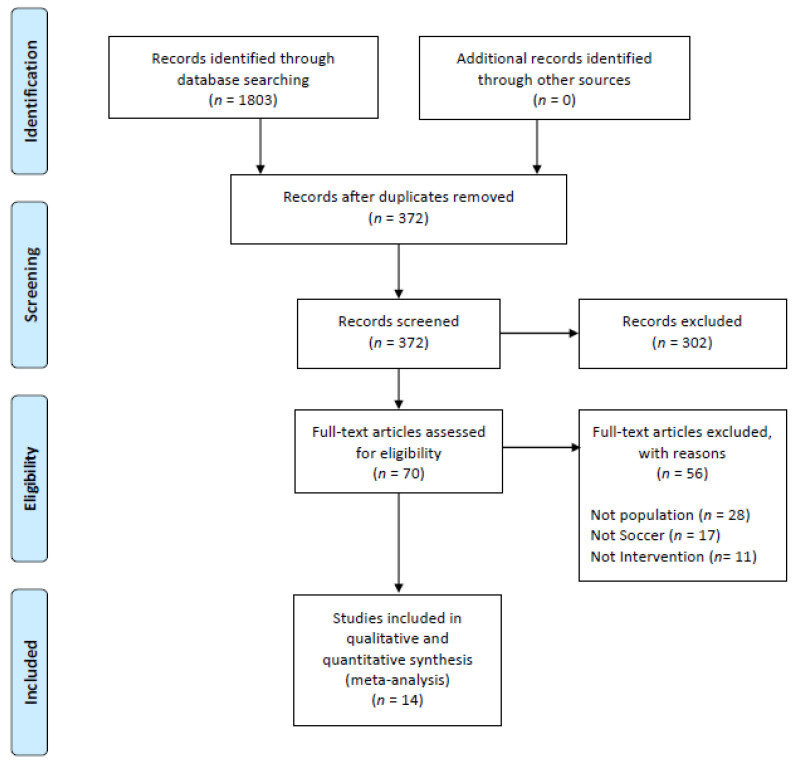
Flow diagram of the selected studies.

The flow diagram in Figure 1 shows the selection process. From a total of 1803 articles, 372 remained after eliminating duplicates. Then, 302 publications were removed because they did not meet the eligibility criteria. Full-text papers (*n* = 70) were assessed for eligibility, with 56 of these being removed for multiple reasons. Finally, 14 studies were included [31,32,33,34,35,36,37,38,39,40,41,42,43,44]. In total, 1643 subjects were evaluated. Table 1 shows the general characteristics of the studies participants. Table 2 and Table 3 show the concrete characteristics and their football intervention.

**Figure 2:**
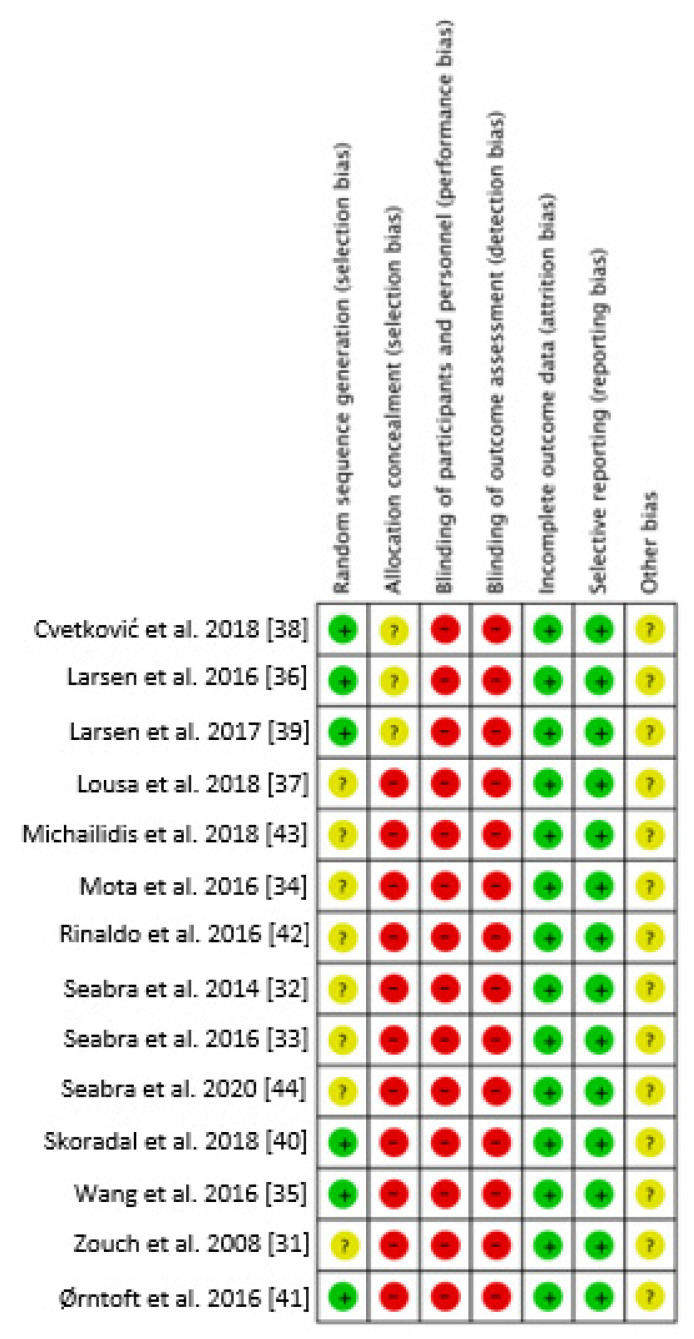
Risk of bias graph of the included studies. **+** Low risk of bias; **-** High risk of bias; **?** Unclear risk of bias [31,32,33,34,35,36,37,38,39,40,41,42,43,44].

**Figure 3:**
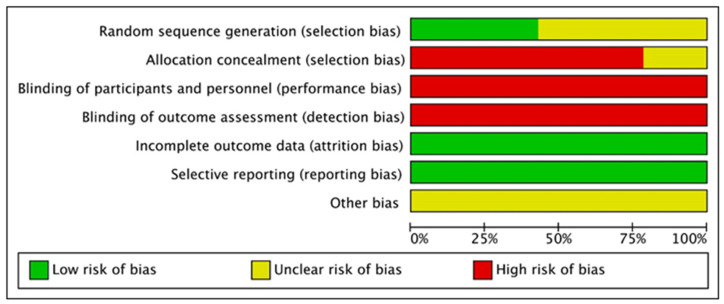
Risk of bias summary for all included studies.

Figure 2 and Figure 3 show the risk of bias representation of the studies analyzed.

**Figure 4:**
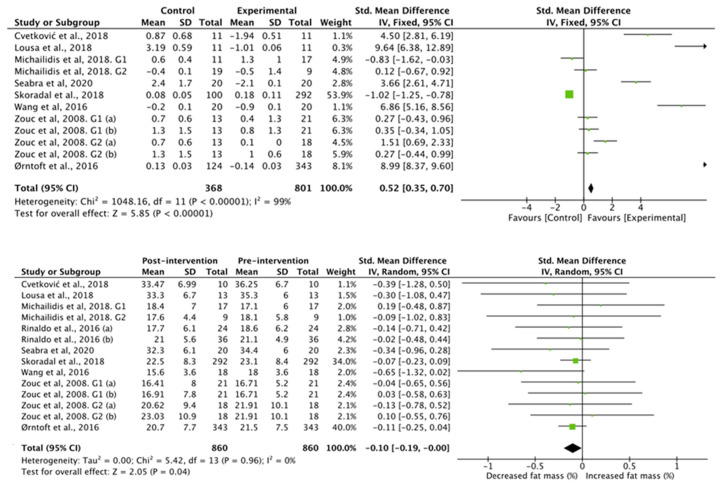
Results of random effects meta-analysis for the control group compared with the experimental group and results post and pre intervention, shown as mean difference and standardized mean difference with 95% CIs for fat mass [31,35,37,38,40,41,42,43,44].

The results obtained in the studies that analyzed fat mass (Figure 4) showed favorable differences for the football group compared to the control group (standardized mean difference of 0.23 (95% CI, 0.22, 0.23), associated with a heterogeneity of I^2^ = 100% and a significance value of *p* < 0.01). Studies with an intervention showed decreases in fat mass after intervention (standardized mean difference −0.81 (95% CI, −1.49, −0.13)), associated with a heterogeneity of I^2^ = 0%, with a significance value of *p* < 0.05).

**Figure 5:**
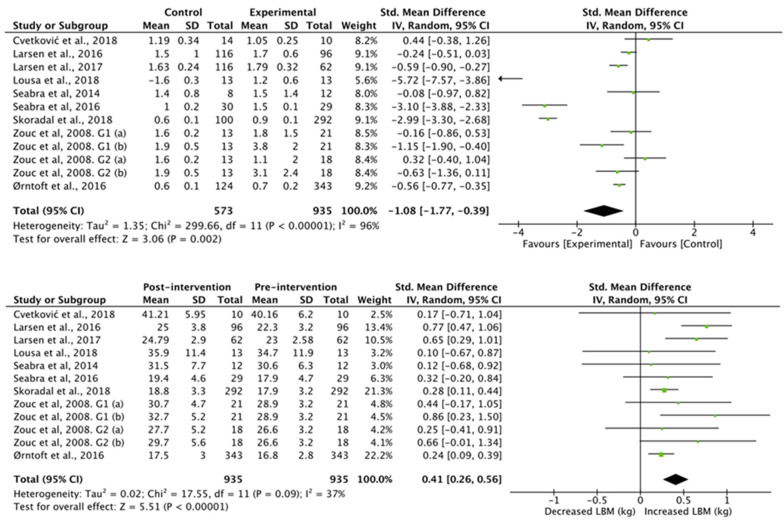
Results of a random effects meta-analysis for control group compared with experimental group and results post and pre intervention, shown as mean difference and standardized mean difference with 95% CIs for lean body mass [31,32,33,36,37,38,39,40,41].

The results obtained in the studies that analyzed lean body mass (Figure 5) showed a higher lean body mass in the football group compared to the control group (standardized mean difference of −0.46 (95% CI, −0.62, −0.29), associated with a heterogeneity of I^2^ = 97% and a significance value of *p* < 0.01). Studies with an intervention showed a significant increase in lean body mass (standardized mean difference of 1.55 (95% CI, 0.96, 2.15), associated with a heterogeneity of I^2^ = 50% and a significance value of *p* < 0.01).

**Figure 6:**
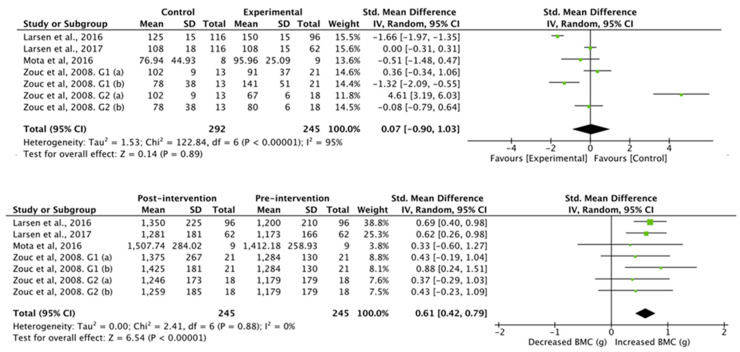
Results of random effects meta-analysis for the control group compared with the experimental group and results post- and pre-intervention, shown as mean difference and standardized mean difference with 95% CIs for bone mineral content [31,34,36,39].

The results obtained in the studies that analyzed bone mineral content (Figure 6) showed no differences between the control group and the football group (standardized mean difference was −7.10 (95% CI, −29.56, 15.36), associated with a heterogeneity of I^2^ = 98% and a significance value of *p* = 0.54). Studies with an intervention showed a significant increase in whole body bone mineral content (standardized mean difference of 117.68 (95% CI, 83.69, 151.67), associated with a heterogeneity of I^2^ = 0% and a significance value of *p* < 0.01).

## 4. Discussion

In the present review, a meta-analysis was conducted to analyze and compare the effects of football practice on body composition (fat mass, muscle mass, and BMC) in children. The selected studies showed significant differences before and after the football intervention in fat mass, lean body mass, and BMC. In addition, significant differences were observed in the comparison between the control and experimental groups in fat mass and lean body mass. Therefore, our analysis showed that football practice in children improves body composition variables of fat mass and lean body mass. However, no significant differences were found between the control and experimental groups in BMC, so more researches on this topic are needed. 

In terms of differences between the control and experimental groups in fat mass, significantly lower levels of fat mass were observed in the children who followed the football intervention. The highest differences were found in studies where the samples were obese children with BMI values higher than 24 kg/m^2^ [37,38,44]. This may indicate that football practice in children with a higher BMI has a more effective impact on the reduction in fat mass due to a significant increase in the energy expenditure, which directly leads to a reduction in body fat [45]. Possible changes in children’s diet and physical activity outside of the study protocols were not considered in the studies, which may also have influenced this reduction in fat mass. However, studies with healthy children [40,43] found that, after the intervention period, the decrease in fat mass was greater in children who did not played football. These studies did not show the intensity of the training sessions which involved 2 h of practice per week. However, it was shown that football practice of at least 3 h per week generates benefits in body composition [46]. This training frequency must reach an adequate threshold of intensity for adaptations to occur [47]. Children who play sport expend higher amounts of energy, but they also tend to consume more calories from unhealthy foods and drinks [48]. The gender of the participants in the intervention may also have an influence. The study by Skoradal [40] showed lower levels of fat mass in the control group whose sample was 50% female. 

Regarding the differences in fat mass before and after intervention, results showed lower levels of fat mass after intervention. Depending on the sample, studies with obese children had values between 2 and 3 kg less fat mass after the intervention [35,37,38,44]. These results are like those found in obese football adults [49]. Interventions of at least 10 and 24 weeks (3 × 60 min sessions/weeks) seem more effective to provide the most significant effect on fat mass reduction. Interventions with longer duration may be contradictory, as children’s own biological growth and development may alter body mass and fat mass levels [50]. Finally, football is mainly an aerobic sport [51], where moderate (71–80 hrmax) or high intensity (81–90 hrmax) activity induces cardiovascular and body composition improvements in adults [52]. In the results of this review, something similar was observed, with interventions that registered an intensity between 70–90 hrmax inducing greater decreases in fat mass. Higher aerobic intensity of the training was positively correlated with higher caloric expenditure and greater loss of fat mass [51].

Regarding the differences obtained between the control groups and the football groups in lean body mass, the results of the different studies analyzed showed higher levels of lean body mass in the football groups (*p* < 0.01). This increase may be related to actions of the game, which involve multiple strengths and the frequent execution of high-intensity actions such as dribbling, shooting, tackling, turning, and jumping (in trainings and matches) [53]. When performing actions, the center of mass is in continuous movement and undergoes abrupt changes in position, with the body supporting its own weight [54]. In contrast, in the study by Cvetković et al. [38] which used a sample of obese children, higher levels of lean body mass were observed in the control group. The reason could be biomechanical, as obese children have higher body weight, but this muscle mass has a more static function compared to the muscle mass of non-overweight children [55]. 

Analysis of the differences before and after the intervention suggests that all of the studies showed higher lean body mass after playing football; however, diet and physical activity outside of football may influence this reduction in lean body mass. However, these results are contradictory to the results found in adults, where there was not a clear increase in lean body mass with football practice [52]. Nevertheless, similarly than with fat mass, differences were found in the studies analyzed. In some interventions, lean body mass increased between 2.7 and 3.8 kg [31,36] and in other studies between 0.7 and 1.5 kg [32,34,38,41]. These inequalities may be attributable to the duration of the interventions, as the studies by Zouch et al. [31] and Larsen et al. [36] are characterized by children realizing 40 weeks of football practice. However, studies with 11-, 12-, or 24-week interventions achieved lower increases in lean body mass. For example, in the study by Zouch et al. [31], G1a and G2a, where the intervention was of 20 weeks, increased by 1.8 and 1.0 kg, respectively. this then increased to 3.8 and 3.1 kg in G1b and G2b, respectively, after 40 weeks of intervention. Therefore, the duration of the intervention is a key factor in the acquisition of lean body mass in children. It is pertinent to remark the energy expenditure, as G1b children had higher levels than G2b children which this may be another cause of the higher lean body mass increase.

Finally, the results obtained in children who played football on the whole-body BMC variable showed no significant differences with respect to the control group. These results suggest that the practice of football does not improve the acquisition of BMC in children. These results are similar to those obtained in the study by Vicente-Rodríguez [56], where an increase was not found in whole-body BMC levels in football players of the same age. The children’s stage of growth and sexual maturity may be decisive for bone mineralization [36] and may be more important than the sport itself in the acquisition of BMC. However, the results obtained before and after the intervention showed significant improvements in BMC. The duration and volume of training was an important factor, as studies with a duration of 40 weeks showed a greater increase in osteogenic development. These results are similar in other sports such as gymnastics, where a positive osteogenic effect was demonstrated in children [57] with high volumes of exercise. Interventions with high volumes of training (40 weeks) at moderate intensity (>70 HRmax) can enhance bone tension and contribute to higher osteogenic development. In addition, a factor that has not been analyzed in the studies considered in this review, and that could be determinant in the acquisition of BMC, is the playing surface. Previous researches showed that higher BMD and BMC values in girls are related to hard playing surfaces with less vertical deformation and a higher energy return [58]. It would be interesting to extend this line of research to football practice for all children. 

The limitations of the studies in this review should be considered, as they may limit the extrapolation of the results. Some of these limitations include lack of information about some of the parameters such as intensity, frequency, duration of the training sessions, and instruments used to measure body composition. There is a lack of control over participants’ involvement in physical/sports activities during the time of the study interventions. Furthermore, the skills and tools used by technicians assessing body composition are unknown. In addition, confidence in participants’ adherence to standard body composition assessment guidelines and research standardization of pre- and post-intervention assessments is unknown. Another aspect to consider is that dietary patterns and energy intake with it macro/micronutrient distribution were not considered in several of the included studies. This is a determinant factor in the improvement of body composition and should therefore be taken into account in future investigations. Being in a physical exercise program may lead to healthier dietary changes. In this sense, future researches should include reliable and well-defined methods to assess body composition and include training load monitoring as well as food intake records.

It has been widely studied that body composition may be the representation of a dietary pattern; increasing the BMI usually is associated with an unhealthy dietary pattern (sweets, fast food, soft drinks, etc.), and reducing it with a healthy dietary pattern (fruits, vegetables, greens, etc.) [59]. However, in the present systematic review with meta-analysis, the results were more focused on the percentage of fat mass, because it is more representative in the athlete population [60]. So, further researches should include a supervised or controlled diet prescribed by an expert dietitian.

## 5. Conclusions

In conclusion, football training in children produces improvements in body composition. It decreases fat mass, especially in overweight children. Interventions at moderate intensity and with a duration from 10 to 24 weeks showed the greatest decreases in fat mass. Lean body mass levels were higher in the football groups, with volume being the key factor, and 40-week interventions seemed to be the most suitable duration to achieve the greatest increases. Finally, osteogenic development was positive and BMC increased after interventions with higher volumes (40 weeks), but this may be influenced by the children’s stage of growth and their dietary pattern. These results make it possible to include the practice of football among the physical activities associated with improvements in health. Parents and educators can include football among the activities for their children and students, especially in those children who are overweight or with obesity problems, as football decreases the levels of fat mass and increases the levels of lean body mass, thus being favorable to prevent or reduce cardiovascular and osteogenic diseases. Nevertheless, the promotion of a good lifestyle should always consider and include adequate nutritional behaviors and good-quality sleep in the children.

Future researches should include monitoring in the children’s diet and nutrition as well as training load, as intensity and volume are key factors in the improvements of the analyzed variables. The results showed more benefits with higher intensity, volume, and duration; thus, the use of global positioning devices could be ideal to control these variables. 

## Figures and Tables

**Table 1:** Main participants characteristics of the studies included in the meta-analysis.

Study and Year		*n*		♂ (%)	Age (years)	BMI (kg/m^2^)
		CG	EG			
Zouch et al. 2008 [31]	Football G1.	13	21	—	12.03 ± 0.83	16.44
	Football G2.		18		11.24 ± 0.65	16.7
Seabra et al. 2014 [32]	Football	12	8	100	10.3 ± 1.8	22.9 ± 2.8
Seabra et al. 2016 [33]	Football	30	29	100	10.5 ± 1.5	23.7± 2.8
Mota et al. 2016 [34]	Football	8	9	100	10.67± 1.8	22.94 ± 3.20
Wang et al. 2016 [35]	Football	20	20	100	9.4 ± 0.7	17.18 ± 1.43
Larsen et al. 2016 [36]	Football	116	96	48	9.3 ± 0.4	17.12
Lousa et al. 2018 [37]	Football	13	13	100	10.6 ± 1.2	24.3 ± 2.9
Cvetković et al. 2018 [38]	Football	14	10	—	11.0± 1.5	25.4 ± 4.1
Larsen et al. 2017 [39]	Football	116	62	—	9-10	16.51
Skoradal et al. 2018 [40]	Football	100	292	50	11.1 ± 0.3	19.3 ± 3.2
Ørntoft et al. 2016 [41]	Football	124	343	55	10-12	18.2 ± 2.7
Rinaldo et al. 2016 [42]	Football a	—	24	100	9	18.1 ± 2.4
	Football b		36		10	19.4 ± 2.9
Michailidis et al. 2018 [43]	G1. Football U10	11	17	—	9.2 ± 0.8	17.8 ± 2.7
	G2. Football U12	19	9		10.8 ± 0.7	18.4 ± 1.5
Seabra et al. 2020 [44]	Football	20	20	—	10.8 ± 1.5	2.3 z-score

*n*: total sample; ♂%, percentage of male children; BMI: Body Mass Index, G1: Group one, G2: Group two.

**Table 2:** General characteristics the studies included in the meta-analysis.

	Group	Exercise	Frequency	Sessions (min)	Duration (Week)	Sessions	Variables	Instruments
			(Week)					
Zouch et al. 2008 [31]	Control	P. E.	2	60	40	80	BMI, BMC, F.M., L.B.M.	Stadiometer, DXA ^1^
	Football 1	Football Training	4 + M	60	20–40	160 + 40		
	Football 2	Football Training	2 + M	60	20–40	80 + 40		
Seabra et al. 2014 [32]	Control	P. E.	2	45–90	20	40	BMI, L.B.M.	Stadiometer, Bioimpedance ^2^, DXA ^1^
	Football	Football Training	4	60–90	20	80		
Seabra et al. 2016 [33]	Control	P. I.	—	—	24	—	BMI, L.B.M.	Stadiometer, Bioimpedance ^2^, DXA ^1^
	Football	Football Training	3	60–90	24	72		
Mota et al. 2016 [34]	Control	P. I.	—		24	—	BMI, BMC	Bioimpedance ^2^, DXA ^1^
	Football	Football Training	4	60–90	24	96		
Wang et al. 2016 [35]	Control	N. A.	—	—	—	—	BMI, F.M.	DXA ^3^
	Football	Football Training	3	60	10	30		
Larsen et al. 2016 [36]	Control	N. A.	—	—	—	—	BMI, BMC, L.B.M.	Stadiometer, Bioimpedance ^4^, DXA ^5^
	Football	Football Training	3	40	40	120		
Lousa et al. 2018 [37]	Control	P. I.	—	—	—	—	BMI, L.B.M., F.M.	Stadiometer, Bioimpedance ^2^, DXA ^1^
	Football	Football Training	3	60–90	24	72		
Cvetković et al. 2018 [38]	Control	P. E.	—	—	—	—	BMI, F.M., L.B.M.	Bioimpedance ^6^
	Football	Football Training	—	—	12	—		
Larsen et al. 2017 [39]	Control	P. E.	—	—	—	—	BMI, BMC, F.M., L.B.M.	Stadiometer, Bioimpedance ^4^, DXA ^5^
	Football	Football Training	1	60	40	40		
Skoradal et al. 2018 [40]	Control	N. A.	—	—	—	—	BMI, F.M., L.B.M.	Bioimpedance ^7^
	Football	Football Training	2	45	11	22		
Ørntoft et al. 2016 [41]	Control	P. E.	—	—	—	—	BMI, F.M., L.B.M.	Stadiometer, Bioimpedance ^7^
	Football	Football Training	2	45	11	22		
Rinaldo et al. 2016 [42]	Football	Football Training	—	—	—	—	BMI, F.M.	Scale, stadiometer Skinfold calliper
	Football	Football Training	4	60	12	48		
Michailidis et al. 2018 [43]	Control U10	P.E.	3	45	40	120	F.M.	Skinfold calliper
	Football U10	Football Training	3 + M	70	40	160		
	Control U12	P.E.	2	45	40	80		
	Football U12	Football Training	3 + M	80	40	160		
Seabra et al. 2020 [44]	Control	P.E.	2	45–90	24	48	F.M.	Bioimpedance ^7^
	Football	Football Training	3	60–90	24	72		

min: minutes; M: match; P. E.: physical education; P. I.: physical inactivity; N. A.: normal activity; L.B.M.: lean body mass; F. M.: fat mass; BMI: body mass index; BMC: bone mineral content; DXA: bone densitometry. *Note:* ^1^ = Hologic QDR 4500A, Hologic Inc., Waltham, MA, USA; ^2^ = Tanita^®^, BC-418MA, Arlington Heights, IL, USA; ^3^ = Prodigy Advance, GE Healthcare Lunar, Madison, WI, USA; ^4^ = Tanita WB-110MA, Tanita, Europe; ^5^ = Lunar Prodigy; E Medical Systems, Madison, Wisconsin, USA; ^6^ = InBody 720; Biospace Co. Ltd., Seoul, Korea; ^7^ = Imbody 230. Biospace, CA, USA.

**Table 3:** Specific characteristics of football practice in the studies included in the meta-analysis.

	Population	Training	Intensity	Instruments	Training Supervision
Zouch et al. 2008 [31]	Healthy	—	—	—	—
Seabra et al. 2014 [32]	Obese	Warm-up (10–20 min), TE and SSG (40–60 min), Cool-down (10 min)	Mean heart rate > 80%HRmax	Polar Team 2 Pro System, (Polar Electro, Kempele, Finland).	P.E. teachers
Seabra et al. 2016 [33]	Obese	Warm-up (10–20 min), TE and SSG (40–60 min), Cool-down (10 min)	Mean heart rate 70–80%HRmax	Polar Team 2 Pro System, (Polar Electro, Kempele, Finland).	P.E. teachers
Mota et al. 2016 [34]	Obese	Warm-up (10–20 min), TE and SSG (40–60 min), Cool-down (10 min)	Progressive increase in intensity according to tolerance	Polar Team 2 Pro System, (Polar Electro, Kempele, Finland).	Researchers
Wang et al. 2016 [35]	Healthy	Warm-up (10 min). DR (10 min), DR and SH (10 min), PA (10 min), 150 and 300-m shuttle run (10 min), Cool-down (10 min)	Progressive increase in intensity according to tolerance	—	Researchers
Larsen et al. 2016 [36]	Healthy	Warm-up (3–5 min), Match 3 vs. 3	Mean heart rate 70–90%HRmax	GPS with accelerometer, RPE	Trainers
Lousa et al. 2018 [37]	Obese	Warm-up (10–20 min), TE and SSG (40–60 min), Cool-down (10 min)	Mean heart rate 70–80%HRmax	Polar Team 2 Pro System, (Polar Electro, Kempele, Finland).	P.E. teachers
Cvetković et al. 2018 [38]	Obese	Warm-up (10–20 min), Match 4 vs. 4, 7 vs. 7 (4 × 8 min, 2 min rest), Cool-down (10 min)	Mean heart rate 70–90%HRmax	Polar Team System H7 (Polar Electro Oy, Kempele, Finland).	Researchers
Larsen et al. 2017 [39]	Healthy	Match 3 vs. 3 (12 min/day)	Mean heart rate 70–90%HRmax	GPS with accelerometer, RPE	Trainers
Skoradal et al. 2018 [40]	Healthy	Activities football skills and match 3 vs. 3, 4 vs. 4 (45 min)	—	—	P.E. teachers
Ørntoft et al. 2016 [41]	Healthy	Activities football skills and match 3 vs. 3, 4 vs. 4 (45 min)	—	—	P.E. teachers
Rinaldo et al. 2016 [42]	Healthy	—	—	—	—
		U10			
		Day 1: TS, C, TA, SSG, COS.			
		Day 2: TS, C, TA SSG SP			
Michailidis et al. 2018 [43]	Healthy	Day 3: TS, CO, TA, SSG	—	—	—
		U12			
		Day 1: TE, C, TA, SSG, AE			
		Day 2: TE, C, TA, SSG, COS			
		Day 3: TE, C, TA, SSG, SP			
Seabra et al. 2020 [44]	Obese	Warm-up (10–20 min), TE and SSG (60 min), Cool-down (10 min)	—	Polar Team 2 Pro System, (Polar Electro, Kempele, Finland)	—

min: minutes; P. E.: physical education; DR: dribbling; SH: shooting; PA: passes; TE: technical skills; C: coordination; TA: tactics; SSG: football games; COS: core strengthening; SP: speed work. Note: Obese = >+2SD (Z-scores (BMI in kg/m^2^)).
